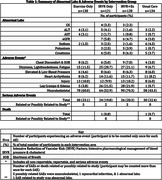# Adverse Events in a Multicenter Randomized Controlled Trial for Dementia Prevention (rrAD study)

**DOI:** 10.1002/alz.092949

**Published:** 2025-01-09

**Authors:** Tristyn Hall, Diana R. Kerwin, Wanpen Vongpatanasin, William Gahan, Jeffrey N. Keller, Ann M Stowe, David C Zhu, Linda Hynan, Munro Cullum, Eric D Vidoni, Jeffrey M Burns, Rong Zhang, Ellen F. Binder

**Affiliations:** ^1^ Institute for Exercise and Environmental Medicine, Texas Health Presbyterian Hospital, Dallas, TX USA; ^2^ Kerwin Medical Center, LLC, Dallas, TX USA; ^3^ UT Southwestern Medical Center, Dallas, TX USA; ^4^ Pennington Biomedical Research Center, Baton Rouge, LA USA; ^5^ University of Kentucky, Lexington, KY USA; ^6^ Michigan State University, East Lansing, MI USA; ^7^ University of Kansas Alzheimer’s Disease Research Center, Fairway, KS USA; ^8^ Washington University in St. Louis, School of Medicine, St. Louis, MO USA

## Abstract

**Background:**

The Exercise and Intensive Vascular Risk Reduction in Preventing Dementia (rrAD study) was a multicenter randomized, controlled trial to determine the effects of moderate to vigorous aerobic exercise training and intensive pharmacological treatment of cardiovascular risk factors on dementia prevention in older adults (NCT02913664). The trial duration was 2 years. We present herein the adverse events (AEs) reported in the rrAD trial. Our objective is to determine whether the trial interventions were associated with higher reports of AEs in older adults aged 60 to 85 who had hypertension, family history of dementia, and/or subjective memory complaints.

**Method:**

513 subjects were randomized to one of four intervention treatment arms: Aerobic Exercise (Ex), Intensive Reduction of Vascular Risk Factors (IRVR), Combined Arm (IRVR+Ex), and Usual Care. The IRVR groups received intensive pharmacological treatment of blood pressure (BP) (SBP<130mmHg) and cholesterol management (atorvastatin, 80mg daily). AEs were categorized by the abnormal lab results and body systems which are likely affected by the trial interventions.

**Result:**

The IRVR arms had slightly higher abnormal lab results of electrolytes (potassium) and creatinine (Table 1) relative to the non‐IRVR arms, which were expected with BP treatment using angiotensin receptor blockers (ARBs) such as, losartan. The IRVR arms also had higher AEs of leg cramps, edema, dizziness, lightheadedness, and fatigue likely reflecting side effects of antihypertensives and/or statin use. The exercise arms had higher AEs in the musculoskeletal system relative to the non‐exercise arms. Overall, the rates of injuries, serious adverse events (SAEs), or death were low, and no noticeable differences were observed among the intervention arms.

**Conclusion:**

Participants in the IRVR arms may report higher AEs related to the side effects of antihypertensives and/or statin use, and in the exercise arms higher AEs related to musculoskeletal events such as muscle pain. The rrAD study showed that it is safe to implement moderate to vigorous aerobic exercise training and intensive cardiovascular risk factor reduction in older adults for dementia prevention.